# Identifying predictors of COVID‐related delays in cancer‐specific medical care

**DOI:** 10.1002/cam4.7183

**Published:** 2024-04-17

**Authors:** Breanna B. Greteman, Natalie J. Del Vecchio, Crystal J. Garcia‐Auguste, Amanda R. Kahl, Brian M. Gryzlak, Elizabeth A. Chrischilles, Mary E. Charlton, Sarah H. Nash

**Affiliations:** ^1^ Department of Epidemiology University of Iowa College of Public Health Iowa City Iowa USA; ^2^ Fred Hutchinson Cancer Center Seattle Washington USA; ^3^ Iowa Cancer Registry University of Iowa Iowa City Iowa USA

**Keywords:** behavioral science, cancer management, clinical observations, epidemiology

## Abstract

**Purpose:**

Evidence of the impact of the COVID‐19 pandemic on cancer prevention and control is growing, but little is known about patient‐level factors associated with delayed care. We analyzed data from a survey focused on Iowan cancer patients' COVID‐19 experiences in the early part of the pandemic.

**Methods:**

Participants were recruited from the University of Iowa Holden Comprehensive Cancer Center's Patients Enhancing Research Collaborations at Holden (PERCH) program. We surveyed respondents on demographic characteristics, COVID‐19 experiences and reactions, and delays in any cancer‐related health care appointment, or cancer‐related treatment appointments. Two‐sided significance tests assessed differences in COVID‐19 experiences and reactions between those who experienced delays and those who did not.

**Results:**

There were 780 respondents (26% response), with breast, prostate, kidney, skin, and colorectal cancers representing the majority of respondents. Delays in cancer care were reported by 29% of respondents. In multivariable‐adjusted models, rural residents (OR 1.47; 95% CI 1.03, 2.11) and those experiencing feelings of isolation (OR 2.18; 95% CI 1.37, 3.47) were more likely to report any delay, where experiencing financial difficulties predicted delays in treatment appointments (OR 5.72; 95% CI 1.96, 16.67). Health insurance coverage and concern about the pandemic were not statistically significantly associated with delays.

**Conclusion:**

These findings may inform cancer care delivery during periods of instability when treatment may be disrupted by informing clinicians about concerns that patients have during the treatment process. Future research should assess whether delays in cancer care impact long‐term cancer outcomes and whether delays exacerbate existing disparities in cancer outcomes.

## Graphical abstract

This manuscript assesses impacts of the COVID‐19 pandemic on cancer‐related medical care among cancer patients receiving care at an academic medical center. We report delays for any cancer care and by treatment type mid‐pandemic and differences between those who experienced delays in care and those who did not in perceptions of social distancing measures, use of social distancing measures, and pandemic‐related concern.

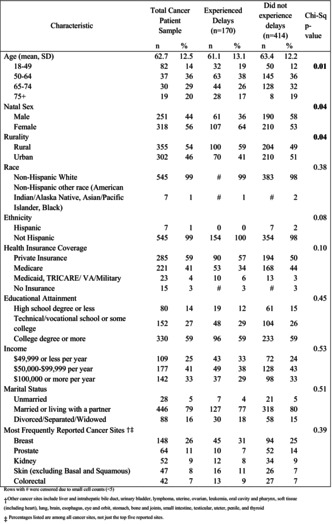

## 1 INTRODUCTION

The impact of COVID‐19 on US healthcare systems was widespread, affecting both general medical care and specialty care.^1^, ^2^ There was particular concern about the impact of the COVID‐19 pandemic on cancer screening, diagnosis, and treatment.^3^, ^4^, ^5^, ^6^, ^7^, ^8^, ^9^, ^10^, ^11^, ^12^, ^13^, ^14^, ^15^, ^16^, ^17^, ^18^, ^19^, ^20^, ^21^ In the early part of the COVID‐19 pandemic (i.e., prior to widespread vaccination), breast cancer imaging, mastectomies, Pap tests, low‐dose computed tomography screening for lung cancer, and colorectal cancer colonoscopy screenings decreased, and there was a reduction in radiological and pathological diagnostic testing.^5^, ^6^, ^7^, ^8^, ^9^ Cancer patients reported experiencing delays across all treatment types, particularly surgery and radiation therapy.^9^, ^10^, ^11^, ^12^, ^16^ Care was disrupted due to facility limitations, and patients canceling appointments out of fear of getting COVID‐19.^5^, ^6^, ^9^ To determine the long‐term impacts of the COVID‐19 pandemic on cancer patients and prepare the healthcare system for any future disruptions, it is important to identify factors that contributed to delays in cancer care.

To address this knowledge gap, we conducted a survey focused on Iowan cancer patients' COVID‐19 experiences in the early part of the pandemic. The purpose of this study was to assess whether cancer‐related health care was delayed in Iowa due to the COVID‐19 pandemic and to identify factors that contributed to delays in cancer care. We expected that survey respondents would report substantial delays in cancer‐related health care. We hypothesized that patients would report more delays in routine check‐ups and surgery than other cancer care types and that health insurance coverage and rurality would be among the strongest predictors of delays in cancer‐related health care.

## 2 METHODS

### 2.1 Data source

Data for this analysis were collected as part of the PERCH (Patients Enhancing Research Collaborations at Holden) Cancer and COVID‐19 Survey at the University of Iowa College of Public Health. Full details on the PERCH Cancer and COVID‐19 survey are being published elsewhere [paper in review]. Briefly, the PERCH Cancer and COVID‐19 Survey was a cross‐sectional e‐mailed Qualtrics survey conducted between September 9, 2020, and October 30, 2020, through partnership with the University of Iowa Holden Comprehensive Cancer Center. A reminder email was sent 2 weeks after initial invitation reminding participants of the survey. Approval for this study was provided by the University of Iowa Institutional Review Board (#202006014).

### 2.2 Study population

PERCH is an opt‐in program for individuals receiving cancer diagnostic testing at the University of Iowa Hospitals and Clinics who are interested in participating in cancer research. Individuals eligible for inclusion in this study were those who enrolled in PERCH after February 1, 2018, with an e‐mail address on file and who were 18 years of age or older. Those excluded from our analytic dataset were those who opted into PERCH after a potential cancer diagnosis and were found to have a negative biopsy result, meaning they did not have cancer. These respondents were identified by a survey item asking patients to indicate whether they ultimately received a cancer diagnosis.

### 2.3 Measures

Data in this analysis were collected from the PERCH survey and subsequent linkage to electronic medical records at the University of Iowa Hospitals and Clinics for collection of clinical data. Survey items were created using a combination of previously validated and newly developed pre‐tested Likert‐type and multiple‐choice items. Skip patterns were implemented for specific questions to reduce the likelihood of erroneously reported data.

Our outcome, delays in cancer care, was measured using a survey item “Were you scheduled for any cancer‐related medical care, surgical care, or follow‐up/surveillance that you had to cancel or reschedule during the COVID‐19 restrictions?” with yes or no response options. Respondents could indicate the types of appointments that were delayed using an item “What did you have to cancel or reschedule?” with response options including routine appointment, screening or cancer follow‐up test, blood test, surgery, chemotherapy, radiation therapy, occupational/physical therapy, and other (please specify). Respondents were able to select any and all that applied.

Demographic characteristics measured by survey items included age in years, sex assigned at birth (male or female, prefer not to answer), race (White, Black, American Indian or Alaska Native, Asian, Native Hawaiian/Pacific Islander, and others), ethnicity (Mexican, Chicano/a, Puerto Rican, Cuban, other Hispanic/Latinx/Spanish origin, or not of Hispanic/Latinx/Spanish origin), household income (categorized into nine levels), education level (less than 8th grade, 8th–11th grade, high school diploma or GED, vocational or technical school, some college, college graduate, and postgraduate), health insurance coverage (employer‐provided, private coverage, Medicare, Medicaid, TRICARE/Veterans Affairs/Military, Alaska Native/Indian Health Service/Tribal Health Services, and others), and marital status (single/never married, married, not married but living with a romantic partner, separated, divorced, widowed). Rurality was measured using Rural–Urban Continuum Codes (RUCCs) and categorizing counties as either rural (codes 4–9) or urban (codes 1–3).

Categories of demographic variables were created for age (18–49, 50–64, 65–74, 75+), natal sex (male, female), race (non‐Hispanic White, non‐Hispanic other race [included American Indian/Alaska Native, Asian/Pacific Islander, and Black participants]), ethnicity (Hispanic, non‐Hispanic), health insurance coverage, educational attainment (high school diploma or less, some college/technical/vocational school, college degree, or more), income ($49,999 or less per year, $50,000–$99,999, $100,000, or more), and marital status (unmarried, married/living with partner, and divorced/separated/widowed). Non‐White race was unable to be categorized with further granularity due to small cell sizes (<5).

We surveyed respondents on the impacts of and experiences related to the COVID‐19 pandemic, including thoughts and behaviors related to social distancing, emotional reactions to the pandemic, and ability to find information on health topics. Thoughts and behaviors about COVID‐19 and social distancing included concern about COVID‐19 (not at all, a little, somewhat, very), receiving emotional, physical, and/or material support in the past 2 weeks (yes/no), whether they lost their job or primary source of income (yes/no), if they had a pay decrease (yes/no), if they lost insurance coverage (yes/no), whether they think social distancing is important (not at all, a little, somewhat, very), whether they felt their cancer history impacted social distancing measures (a great deal, a moderate amount, a little, none at all), whether COVID‐19 impacted their cancer care, whether COVID‐19 disrupted their care, felt social distancing kept them safe, and felt social distancing was easy to do (all of which had response options strongly disagree, disagree, neutral, agree, strongly agree).

Emotional reactions to the pandemic included items on whether the respondent felt their cancer history impacted social distancing measures, COVID‐19 impacted their cancer care, COVID‐19 disrupted their care, felt social distancing kept them safe, felt social distancing was easy to do, were nervous/anxious/on edge, were anxious about getting COVID‐19, felt more vulnerable to COVID‐19 due to their cancer history, were worried about dying from COVID‐19, were worried about friends/family dying from COVID‐19, experienced feelings of social isolation or loneliness, had difficulty or inability to work as usual, experienced financial difficulties, and felt their healthcare team shared adequate information on prevention, protection, or care for COVID‐19 (all of which had response options strongly disagree, disagree, neutral, agree, strongly agree). Items based on their most recent search for information asked about effort needed to find information, frustration when searching for information, concern about the quality of information, and thinking that the information was hard to understand (strongly disagree, disagree, agree, strongly agree).

### 2.4 Statistical analysis

All data were analyzed using SAS 9.4 software (SAS Institute, Cary, NC). We calculated frequencies for all variables and used Chi‐square tests to assess differences in demographic characteristics between those who reported delays in care compared to those who reported no delays in care. We used univariate logistic regression to individually assess potential predictors of delays in cancer care. Multivariable‐adjusted logistic regression models were used to determine independent predictors of delays in cancer care. Variable selection for predictive modeling was based on a combination of univariate model significance, correlation between variables (i.e., choosing between variables with high correlation), and scientific reasoning for variables that could feasibly result in care delays.

Outcomes in these models were delays in care, either reported for any type of appointment (routine appointment, screening, blood test, surgery, chemotherapy, radiation therapy, physical/occupational therapy, and others) or specific to cancer treatment (surgery, chemotherapy, radiation therapy). Covariates (predictors) in the models included age (<65 year [referent] vs. ≥65 year), marital status (married [referent] vs. unmarried), income ($50,000 or more per year [referent] vs. $49,999 or less per year), insurance (three variables for private insurance [referent] vs. Medicare, Medicaid/TRICARE/VA/Military, and no insurance), race (White [referent] vs. non‐White), education (two variables for college degree or more [referent] vs. some college/technical/trade school, high school degree or less), and impact and emotional response variables (disagree/strongly disagree/neutral [referent] vs. agree/strongly agree). All significance tests were two‐sided with *p* < 0.05 considered statistically significant in all analyses.

## 3 RESULTS

The survey yielded a 26% response with 780 respondents. Of these, 123 were found to not have cancer and were excluded, resulting in 657 respondents in our analytic sample of patients who opted into the PERCH registry and had an email on file after February 2018. Among the analytic sample, 130 patients were diagnosed in 2003–2017 and 443 were diagnosed in 2018–2020, the mean age was 62.7 years, 56% were female, 79% were married or living with a partner, 54% were rural, 99% were non‐Hispanic White, 59% had private insurance coverage, 3% were uninsured, and 59% had college degree or higher educational attainment (Table 1). The majority of respondents were concerned about COVID‐19 (92%), felt their cancer history impacted their social distancing measures (83%), thought social distancing was easy (70%), keeping them safe (78%), and important (98%) (Table 2). Nearly two‐thirds of respondents were worried about dying from COVID‐19, but less than half of respondents were nervous/anxious/on edge (34%), were anxious about getting COVID‐19 (45%), felt isolated (23%), had difficulty or inability to work as usual (22%), and experienced financial difficulties (14%).

**TABLE 1:** Demographic characteristics of University of Iowa Holden Comprehensive Cancer Center PERCH survey respondents, stratified by those who experienced and who did not experience cancer care delays (*n* = 657), 2020.

Characteristic	Total cancer patient sample		Experienced delays (*n* = 170)		Did not experience delays (*n* = 414)		Chi‐Sq *p*‐value
	*n*	%	*n*	%	*n*	%	
Age (mean, SD)	62.7	12.5	61.1	13.1	63.4	12.2	
18–49	82	14	32	19	50	12	**0.01**
50–64	37	36	63	38	145	36	
65–74	30	29	44	26	128	32	
75+	19	20	28	17	8	19	
Natal sex							
Male	251	44	61	36	190	58	**0.04**
Female	318	56	107	64	210	53	
Rurality							
Rural	355	54	100	59	204	49	**0.04**
Urban	302	46	70	41	210	51	
Race							
Non‐hispanic white	545	99	#	99	383	98	0.38
Non‐Hispanic other race (American Indian/Alaska Native, Asian/Pacific Islander, Black)	7	1	#	1	#	2	
Ethnicity							
Hispanic	7	1	0	0	7	2	0.08
Not hispanic	545	99	154	100	354	98	
Health insurance coverage							
Private insurance	285	59	90	57	194	50	0.10
Medicare	221	41	53	34	168	44	
Medicaid, TRICARE/ VA/Military	23	4	10	6	13	3	
No insurance	15	3	#	3	#	3	
Educational attainment							
High school degree or less	80	14	19	12	61	15	0.45
Technical/vocational school or some college	152	27	48	29	104	26	
College degree or more	330	59	96	59	233	59	
Income							
$49,999 or less per year	109	25	43	33	72	24	0.53
$50,000–$99,999 per year	177	41	49	38	128	43	
$100,000 or more per year	142	33	37	29	98	33	
Marital status							
Unmarried	28	5	7	4	21	5	0.51
Married or living with a partner	446	79	127	77	318	80	
Divorced/Separated/Widowed	88	16	30	18	58	15	
Most frequently reported cancer sites^a^ ^,^ ^b^							
Breast	148	26	45	31	94	25	0.39
Prostate	64	11	10	7	52	14	
Kidney	52	9	12	8	34	9	
Skin (excluding Basal and Squamous)	47	8	16	11	26	7	
Colorectal	42	7	13	9	27	7	

*Note*: Rows with # were censored due to small cell counts (<5). Bold values are two‐sided with *p* < 0.05 considered statistically significant.

^a^ Other cancer sites include liver and intrahepatic bile duct, urinary bladder, lymphoma, uterine, ovarian, leukemia, oral cavity and pharynx, soft tissue (including heart), lung, brain, esophagus, eye and orbit, stomach, bone and joints, small intestine, testicular, uteter, penile, and thyroid.

^b^ Percentages listed are among all cancer sites, not just the top five reported sites.

**TABLE 2:** Differences in responses to and impacts of the COVID‐19 pandemic between those who did and did not experience treatment delays, University of Iowa Holden Comprehensive Center PERCH survey respondents (*n* = 657), 2020.

Characteristic	Total cancer patient sample (*n* = 657)		Experienced delays (*n* = 170)		Did not experience delays (*n* = 414)		Chi‐Sq *p*‐value
	*n*	%	*n*	%	*n*	%	
Was concerned (a little, somewhat, very) Regarding COVID‐19	571	92	160	95	383	93	0.55
Agree/strongly agree with feeling their cancer history impacted social distancing measures	512	83	149	88	343	83	0.15
Agree/Strongly Agree with feeling COVID‐19 impacted cancer care	107	18	39	21	68	17	0.06
Agree/Strongly Agree with feeling COVID‐19 disrupted cancer care	87	16	63	39	24	6	**<0.0001**
Agree/Strongly Agree with thinking social distancing keeps them safe	475	78	129	76	328	79	0.41
Agree/Strongly Agree with thinking social distancing is easy to do	396	70	109	66	269	71	0.27
Agree/Strongly Agree that social distancing is important	595	98	166	98	405	98	0.90
Received support (e.g., emotional, material, or financial) in previous 2 weeks	331	55	73	43	221	54	0.58
Lost job or primary source of income	14	2	7	4	7	2	0.08
Had a pay decrease	24	4	5	3	17	4	0.62
Lost insurance coverage	0	0	0	0	0	0	‐
Indicate your agreement with the following statements: Since the onset of COVID‐19…							
I have felt nervous, anxious, or on edge	204	34	77	46	120	30	**0.0001**
I have felt anxious about getting COVID‐19	266	45	91	55	168	42	**0.004**
I feel more vulnerable to COVID‐19 due to my cancer history	257	44	79	48	167	42	0.18
I am worried about dying from COVID‐19	401	68	123	74	263	65	**0.04**
I am worried about their family or friends dying from COVID‐19	342	58	100	60	232	57	0.50
I have experienced feelings of isolation or loneliness	133	23	57	35	72	18	**<0.0001**
I have experienced difficulty or inability to work as usual	123	22	46	29	72	19	**0.007**
I have experienced financial difficulties	81	14	33	20	45	11	**0.006**
In your most recent search for information about health or medical topics, how much do you agree or disagree with each of the following statements?							
Agree/Strongly Agree that it took a lot of effort to get needed information	120	22	43	27	77	20	0.08
Agree/Strongly Agree with being frustrated during search for information	103	19	36	23	67	18	0.13
Agree/Strongly Agree with feeling concern about the quality of information	167	32	48	31	119	32	0.95
Agree/Strongly Agree with feeling that information from health or medical topic searches was hard to understand	121	23	37	24	84	23	0.65

*Note*: Bold values are two‐sided with *p* < 0.05 considered statistically significant.

### 3.1 Cancer care delays

Almost one‐third (29%, *n* = 170) of respondents reported delays in their cancer care due to the COVID‐19 pandemic. Among those that reported delays, 38% reported delays for routine appointments, 23% for screenings, 15% for blood tests, 6% for surgery, 3% for chemotherapy, 3% for physical/occupational therapy, and 1% for radiation therapy. Ten percent of delayed appointments were unable to be attributed to one of these categories and were listed as “other” (Table 3). Respondents who experienced delays were younger (*p* = 0.01), had a higher proportion of natal sex females (64% vs. 53%, *p* = 0.04), and had a higher proportion of rural residents (*p* = 0.04) compared to those who did not experience delays. Differences were not observed for race, ethnicity, health insurance coverage, educational attainment, income, or marital status. Among respondents who experienced delays, the mean age was 61.1 years, 64% were female, 77% were married or living with a partner, 59% were rural, 99% were non‐Hispanic White, none identified as Hispanic, 57% had private insurance coverage, 3% were uninsured, and 59% had college degree or higher educational attainment. Among respondents with no delays, the mean age was 63.4 years, 53% were female, 80% were married or living with a partner, 49% were rural, 98% were non‐Hispanic White, 50% had private insurance coverage, 3% were uninsured, and 59% had college degree or higher educational attainment.

**TABLE 3:** Types of cancer care appointments delayed among University of Iowa Holden Comprehensive Cancer Center PERCH Survey respondents that identified cancer‐related health care delays during COVID‐19 restrictions (*N* = 170), 2020.

Type of appointment that was canceled/delayed^a^	*n* (%)
Routine appointment	106 (38)
Screening or cancer follow‐up test	63 (23)
Blood test	41 (15)
Surgery^b^	18 (6)
Chemotherapy^b^	10 (<5)
Radiation therapy^b^	<5 (<5)
Physical or occupational therapy	9 (<5)
Other^c^	29 (10)

^a^ Respondents were able to select more than one appointment type.

^b^ Variables included in the “treatment delays” variable.

^c^ Responses in the “other” category are those that were unable to be attributed to other appointment type categories.

### 3.2 Cancer care delays and perspectives on COVID‐19

Respondents who experienced delays more frequently felt that COVID‐19 disrupted their care (39% vs. 6%, *p* < 0.0001), felt nervous/anxious/on edge (46% vs. 30%, *p* = 0.0001), felt anxious about getting COVID‐19 (55% vs. 42%, *p* = 0.004), worried about dying from COVID‐19 (74% vs. 65%, *p* = 0.04), felt isolated or lonely (35% vs. 18%, *p* < 0.0001), experienced difficulty or inability to work as usual (29% vs. 19%, *p* = 0.007), and experienced financial difficulties (20% vs. 11%, *p* = 0.006).

### 3.3 Predictors of cancer care delays

In univariate models, statistically significant predictors of any care delays were living in a rural area (OR = 1.47, 95% CI: (1.03, 2.11)), female natal sex (OR = 1.59, 95% CI: (1.10, 2.30)), Medicare insurance coverage (OR = 0.66, 95% CI: (0.45, 0.97)), perceptions of COVID‐19 disrupting their cancer care (OR = 9.94, 95% CI: (5.91, 16.72)), feeling nervous, anxious, or on edge (OR = 2.04, 95% CI: (1.41, 2.96)), feeling anxious about getting COVID‐19 (OR = 1.70, 95% CI: (1.18, 2.44)), worrying about dying from COVID‐19 (OR = 1.51, 95% CI: (1.01, 2.26)), experiencing feelings of social isolation or loneliness (OR = 2.41, 95% CI: (1.60, 3.63)), experiencing difficulty or inability to work as usual (OR = 1.80, 95% CI: (1.17, 2.76)), and experiencing financial difficulties (OR = 1.98, 95% CI: (1.21, 3.24)) (Table 4).

**TABLE 4:** Univariate logistic regression models identifying predictors of delays in any cancer‐related health care appointments and for cancer treatment appointments among University of Iowa Holden Comprehensive Cancer Center PERCH Survey respondents (*n* = 657), 2020.

Characteristics	Predictors of any cancer care delays			Predictors of treatment delays^a^		
	OR	95% CI	*p*‐value	OR	95% CI	*p*‐value
Rural (ref. urban)	1.47	1.03, 2.11	**0.04**	0.71	0.32, 1.58	0.40
Aged 65 years or older (ref. younger than 65)	0.74	0.52, 1.06	0.10	0.80	0.35, 1.82	0.60
Unmarried (ref. married)	1.17	0.75, 1.82	0.48	0.67	0.24, 1.90	0.45
Female Natal Sex (ref. male)	1.59	1.10, 2.30	**0.01**	0.65	0.29, 1.46	0.30
$49,999 Income or less per year (ref. $50,000 or more)	1.26	0.79, 2.01	0.33	1.08	0.38, 3.06	0.89
Health insurance coverage						
Medicare Coverage (ref. private insurance)	0.66	0.45, 0.97	**0.03**	1.44	0.63, 3.31	0.39
Medicaid or VA Coverage (ref. private insurance)	1.93	0.83, 4.49	0.13	1.23	0.25, 6.12	0.80
Educational attainment						
Some college/technical school education (ref. college degree or more)	0.92	0.72, 1.19	0.55	1.15	0.66, 2.03	0.62
High school education or less (ref. college degree or more)	0.86	0.51, 1.42	0.55	1.33	0.43, 4.10	0.62
Reactions to/Experiences with COVID‐19						
Was concerned about COVID‐19 in their community	1.25	0.59, 2.62	0.55	‐	‐	‐
Felt cancer history impacted personal social distancing measures	1.47	0.87 2.48	0.15	‐	‐	‐
Felt COVID‐19 impacted cancer care	1.52	0.98, 2.37	0.06	2.00	0.84, 4.76	0.12
Felt COVID‐19 disrupted cancer care	9.94	5.91, 16.72	**<0.0001**	2.04	0.90, 4.65	0.09
Felt social distancing kept them safe	0.84	0.54, 1.28	0.41	1.23	0.46, 3.27	0.68
Felt social distancing was easy to do	0.80	0.54, 1.19	0.27	0.95	0.41, 2.21	0.91
Thought social distancing was important/very important	1.09	0.29, 4.17	0.90	‐	‐	‐
Received support (e.g., emotional, material, or financial) in previous 2 weeks	1.11	0.77, 1.59	0.58	1.30	0.57, 2.96	0.53
Lost their job or primary source of income	2.50	0.86, 7.23	0.09	‐	‐	‐
Had a pay decrease	0.77	0.28, 2.13	0.62	‐	‐	‐
Were nervous, anxious, or on edge	2.04	1.41, 2.96	**0.0002**	0.80	0.36, 1.81	0.60
Were anxious about getting COVID‐19	1.70	1.18, 2.44	**0.004**	1.44	0.63, 3.26	0.39
Felt more vulnerable to COVID‐19 due to their cancer history	1.28	0.89, 1.84	0.18	1.43	0.64, 3.19	0.39
Worried about dying from COVID‐19	1.51	1.01, 2.26	**0.04**	1.42	0.54, 3.76	0.48
Worried about their family or friends dying from COVID‐19	1.14	0.79, 1.64	0.50	2.99	1.14, 7.80	**0.03**
Experienced feelings of social isolation/loneliness	2.41	1.60, 3.63	**<0.0001**	1.20	0.52, 2.74	0.67
Had difficulty or inability to work as usual	1.80	1.17, 2.76	**0.007**	1.76	0.75, 4.12	0.19
Experienced financial difficulties	1.98	1.21, 3.24	**0.006**	3.97	1.66, 9.50	**0.002**
Felt their healthcare team shared adequate information on prevention, protection, or care for COVID‐19	0.88	0.54, 1.43	0.60	1.07	0.37, 3.11	0.90
Felt that it took a lot of effort to get needed information when searching for health or medical topics	1.47	0.96, 2.26	0.08	0.91	0.36, 2.32	0.84
Felt frustrated when searching for health or medical topics	1.42	0.90, 2.25	0.13	1.40	0.56, 3.52	0.47
Was concerned about the quality of information when searching for health or medical topics	0.99	0.65, 1.48	0.95	0.68	0.27, 1.74	0.42
Felt that information from health or medical topic searches was hard to understand	1.11	0.71, 1.73	0.65	0.91	0.34, 2.47	0.85

*Note*: Variables with “‐ “were unable to be used in multivariable models due to convergence issues. Bold values are two‐sided with *p* < 0.05 considered statistically significant.

^a^ Treatment delays include postponed or canceled surgery, chemotherapy, and radiation therapy appointments.

In univariate models, statistically significant predictors of cancer treatment care delays included worrying about friends or family dying from COVID‐19 (OR = 2.99, 95% CI: (1.14, 7.80)) and experiencing financial difficulties (OR = 3.97, 95% CI: (1.66, 9.50)) (Table 4).

In multivariable models, statistically significant predictors of any care delays were rurality (aOR = 1.56, 95% CI: (1.05, 2.33)) and experiencing feelings of isolation/loneliness (aOR = 2.18, 95% CI: (1.37, 3.47)) (Table 5). The only statistically significant predictor of treatment delays in multivariable models was experiencing financial difficulties (aOR = 5.72, 95% CI: (1.96, 16.67)).

**TABLE 5:** Multivariable‐adjusted logistic regression models identifying predictors of delays in any cancer‐related health care appointments and for cancer treatment appointments among University of Iowa Holden Comprehensive Cancer Center PERCH Survey respondents (*n* = 657), 2020.

	Predictors of any cancer care delays			Predictors of treatment delays^a^		
	OR	95% CI	*p*‐value	OR	95% CI	*p*‐value
Rurality (ref. urban)						
Rural	1.56	1.05, 2.33	**0.03**	0.80	0.33, 1.91	0.61
Age (ref. younger than 65 years)						
65 years or older	1.44	0.80, 2.61	0.23	1.21	0.45, 3.22	0.71
Natal sex (ref. Male)						
Female	1.46	0.96, 2.23	0.08	0.55	0.22, 1.37	0.20
Insurance coverage (ref. Private insurance)						
Medicare	0.64	0.35, 1.17	0.15	‐	‐	‐
Medicaid and/or VA/military coverage	1.26	0.48, 3.30	0.64	0.58	0.09, 3.77	0.57
Reactions to/experiences with COVID‐19						
Lost job or income (ref. No)	1.54	0.45, 5.13	0.49	‐	‐	‐
Experienced anxiety regarding COVID‐19 infection (ref. disagree/strongly disagree/neutral)	1.44	0.96, 2.18	0.08	1.03	0.41, 2.55	0.96
Experienced feelings of isolation (ref. disagree/strongly disagree/neutral)	2.18	1.37, 3.47	**0.001**	1.07	0.42, 2.73	0.88
Experienced financial difficulties (ref. disagree/strongly disagree/neutral)	1.56	0.87, 2.78	0.13	5.72	1.96, 16.67	**0.001**
Frustrated with effort needed to find information on health topics (ref. disagree/strongly disagree/neutral)	1.13	0.71, 1.80	0.60	0.78	0.27, 2.27	0.65

*Note*: Variables with “‐”were unable to be used in multivariable models due to convergence issues. Bold values are two‐sided with *p* < 0.05 considered statistically significant.

^a^ Treatment delays include postponed or canceled surgery, chemotherapy, and radiation therapy appointments.

## 4 DISCUSSION

In this study, only 29% (170 of 585) of Iowa cancer patients reported they experienced delays to their cancer care in the early part of the pandemic, with routine appointments being the most frequently reported type of appointment cancelation (38% of appointments canceled). In multivariable‐adjusted models, factors associated with any cancer‐related appointment delays were rurality and experiencing feelings of loneliness; the only statistically significant factor associated with treatment delays was experiencing financial difficulties. We did not find that health insurance coverage, concern about the COVID‐19 pandemic, and/or anxiety about catching or dying from COVID‐19 were statistically significantly associated with healthcare delays in multivariable models. These findings indicate that while most Iowans did not experience disturbances to their care, those that did were more impacted by proximity to care and financial concerns than concerns about COVID‐19 itself. We were unable to determine whether this lack of concern was attributable to healthcare practices successfully mitigating fears (e.g., mandatory mask‐wearing, restricting visitors to encourage physical distancing) or a general lack of concern about the COVID‐19 pandemic in this mostly rural sample. Nevertheless, our findings indicate that barriers to care during the COVID‐19 pandemic mirrored those faced during non‐pandemic times, suggesting that the pandemic may have widened already existing disparities.

Our findings are consistent with other studies on cancer treatment delays in the early part of the pandemic. In two qualitative studies exploring the experience of cancer patients during the pandemic, delays in care presented as main themes.^23^, ^24^ The frequency of individuals reporting delays ranged from 18% to 47% in two quantitative studies conducted in single centers.^25^, ^26^ In studies stratifying delays by treatment type, delays in follow‐up and patient management ranged from 44% to 74% while individuals reporting delays in surgery were around 14%.^7^, ^12^ Delays in care related to the COVID‐19 pandemic were not specific to the U.S. healthcare experience: our results were also consistent with studies outside of the U.S.^10^, ^11^, ^12^, ^13^, ^14^, ^15^, ^21^, ^27^, ^28^, ^29^, ^30^, ^31^, ^32^, ^33^ Two studies in the Philippines and Kenya reported between 33% and 42% of patients experienced general delays in care.^27^ In studies that stratified by treatment type, studies from Germany, Austria, Spain, and the Netherlands reported between 21% and 31% of patients experiencing delays for follow‐up visits;^15^, ^28^, ^29^, ^30^ studies from India, Germany, and China reported 9% to 52% patients experiencing cancer surgery delays;^9^, ^10^, ^29^, ^32^ and, studies from Spain, Italy, India, and China reported between 23% to 38% patients reporting delays in radiotherapy.^8^, ^28^, ^32^, ^33^ Additionally, a majority of patients in three studies reported differences in median time to surgery during the pandemic compared to before the pandemic in the U.K. and Greece.^34^, ^35^ This consistency demonstrated in national and global findings indicates the importance of addressing cancer care delays and in opportunities for learning from solutions identified in different global contexts.

In our study, those who experienced delays were typically younger in age, more often female (perhaps due to the large number of breast cancer patients present in our sample), more often rural, and experienced greater financial difficulties. The interplay between these factors is potentially important, as rurality and low socioeconomic status are predictors of worsened cancer outcomes.^36^, ^37^, ^38^, ^39^, ^40^ Our findings, along with findings from other studies, may imply that the COVID‐19 pandemic could be widening existing disparities in early detection and treatment that exist by race and socioeconomic status, which in turn is likely to affect cancer outcomes.^14^, ^41^, ^42^, ^43^ There is a need for long‐term monitoring of cancer surveillance data to track whether disparities maintain or continue to widen for individuals living in rural areas and of low socioeconomic status. This long‐term monitoring should also prioritize a more racially diverse sample, as there was a lack of racial diversity in our study due to our mostly non‐Hispanic White sampling frame.

This study has several limitations that warrant consideration when interpreting its findings. This study was cross‐sectional; therefore, we were unable to assess temporality in the relationship between COVID‐19 patient experiences and care delays. Future studies temporally assessing delays to cancer care due to large‐scale events are important for identifying mechanisms for delays and opportunities to intervene and minimize said delays. Additionally, our 26% response rate increases the potential of results being impacted by selection bias; those who responded may have different demographic and clinical characteristics than those who did not. However, the goal of this study was to assess COVID‐19 delays at a particular academic medical center. Due to only living cancer patients being able to participate in the survey, there is a risk for results being impacted by survivor bias; however, it is unlikely that delays in patients who survived to participate in the survey would be different than delays among those who did not survive until the survey.

The sample of those invited to participate in the survey were those who were diagnosed or treated at an academic medical center, which may minimize the generalizability of these results. As respondents in this survey were diagnosed and treated at this one location, we could not examine variability in delays by facility, but rather we examined personal or social factors that may be associated with healthcare delays. Prior studies suggest that facility characteristics, specifically organizational‐level factors such as centralization, patient outreach systems, and facility‐level factors such as reductions in provider/service availability, are important predictors of cancer care delays and, thus, important to intervene on when attempting to minimize disparities.^11^, ^22^

This study illuminates the experiences of patients at a Midwest cancer center during the early part of COVID‐19. Since cancer treatment and follow‐up appointments are integral parts of preventing poor cancer outcomes, these findings provide important insight into the experiences of cancer patients during the part of the pandemic that may have impacted longer‐term outcomes among Iowa patients. We found that rurality and experiencing social isolation were the factors most strongly associated with delays in any type of cancer care, and experiencing financial difficulties was most strongly associated with delays in cancer treatment appointments. These findings can inform cancer care delivery in future destabilizing events that may impact cancer care, and inform potential areas for improvement during times when health systems are not stressed, with the goal of alleviating impacts during such destabilizing events. Future research should also assess whether delays in cancer care impact long‐term cancer outcomes.

## AUTHOR CONTRIBUTIONS

**Breanna B. Greteman:** Conceptualization (equal); data curation (equal); formal analysis (lead); investigation (equal); methodology (lead); software (equal); visualization (lead); writing – original draft (lead). **Natalie J. Del Vecchio:** Conceptualization (equal); data curation (equal); investigation (equal); writing – review and editing (equal). **Crystal J. Garcia‐Auguste:** Conceptualization (equal); writing – review and editing (equal). **Amanda R. Kahl:** Data curation (equal); formal analysis (supporting); validation (equal); writing – review and editing (equal). **Brian M. Gryzlak:** Data curation (equal); investigation (equal); project administration (lead); software (equal); validation (equal); writing – review and editing (equal). **Elizabeth A. Chrischilles:** Conceptualization (equal); funding acquisition (equal); investigation (equal); resources (equal); supervision (equal); writing – review and editing (equal). **Mary E. Charlton:** Conceptualization (equal); funding acquisition (equal); investigation (equal); resources (equal); supervision (equal); writing – review and editing (equal). **Sarah H. Nash:** Conceptualization (equal); investigation (equal); supervision (equal); writing – original draft (supporting).

## FUNDING INFORMATION

The project was supported in part by the University of Iowa Holden Comprehensive Cancer Center 3P30CA086862 and COVID‐19 Supplement Grant 3P30CA086862‐19S5. The work of BBG, ARK, SHN, and MEC are additionally supported by the National Institutes of Health‐National Cancer Institute contract number HHSN261201800012I/HHSN26100001.

## CONFLICT OF INTEREST STATEMENT

The authors in this manuscript have no conflicts of interest to disclose.

## ETHICS STATEMENT

This study was determined to be exempt from full board review by the Institutional Review Board at the University of Iowa (IRB ID #201910790) and was conducted in line with the ethical standards described in the 1964 Helsinki Declaration and its later amendments.

## Data Availability

Deidentified data can be made available upon request.
